# *In-Vitro* Study on the Antibacterial and Antioxidant Activity of Four Commercial Essential Oils and *In-Situ* Evaluation of Their Effect on Quality Deterioration of Pacific White Shrimp (*Litopenaeus vannamei*) during Cold Storage

**DOI:** 10.3390/foods11162475

**Published:** 2022-08-17

**Authors:** Yun-Fang Qian, Ting Lin, Xiao Liu, Jiao Pan, Jing Xie, Sheng-Ping Yang

**Affiliations:** 1 College of Food Science & Technology, Shanghai Ocean University, Shanghai 201306, China; 2 Shanghai Engineering Research Center of Aquatic Product Processing & Preservation, Shanghai 201306, China; 3 Department of Food and Nutrition, University of Helsinki, 00014 Helsinki, Finland; 4 Henan Key Laboratory of Cold Chain Food Quality and Safety Control, Zhengzhou University of Light Industry, Zhengzhou 450001, China

**Keywords:** oregano essential oil, clove leaf essential oil, melanosis ratio, protein degradation, TCA-soluble peptides, mechanism

## Abstract

The antioxidant and antibacterial properties of four essential oils (oregano essential oil (OEO), tea tree essential oil (TTEO), wild orange essential oil (WOEO), and clove leaf essential oil (CLEO)) were determined. The *in-vitro* experiment indicated that CLEO had the highest total phenolic content and DPPH scavenging activity, and OEO displayed the highest antibacterial effect, so they were applied to maintain the quality of shrimp for further study. *In-situ* study, the total viable counts of shrimp were inhibited from 9.05 log CFU/g to 8.18 and 8.34 log CFU/g by 2% of OEO and CLEO treated alone on 10 d. The melanosis ratio was also retarded from 38.16% to 28.98% and 26.35% by the two essential oils. The inhibitory effects of OEO and CLEO on the increase of PPO activity, weight loss, and TCA-soluble peptides, and the decreasing tendency of whiteness, the contents of myofibrillar and sarcoplasmic proteins were also founded. The samples treated with 1% OEO + 1% CLEO had better quality than those treated alone. Therefore, the combination of OEO and CLEO had a synergistic effect, which displayed the highest efficiency to prevent the melanosis, bacterial growth, and protein hydrolysis of shrimp.

## 1. Introduction

Essential oils (EO) are important plant extracts and have attracted much interest from scientists for their various biological activities, including anticancer, anti-obesity, antispasmodic effects, etc. [1,2,3]. They are also potential to replace chemical preservatives in the field of food preservation due to their antibacterial, antiviral, antifungal, and antioxidant effects [4,5,6]. Studies have demonstrated the efficiency of EOs to inhibit the growth of pathogens and spoilers, and extend the shelf life of seafoods [7,8,9]. Furthermore, as they are derived from plants, they are generally recognized as safe (GRAS) by the United States Food and Drug Administration (USFDA) [4].

The effectiveness of EOs on inhibiting bacterial growth and prolonging the shelf-life of various foods are proven [10,11,12]. However, EOs are very complex, because they are composed of at least 50 components. It is difficult to predict the susceptibility of a microorganism to a kind of EOs [11]. Therefore, it is necessary to study the formula of the extracts on different food products individually.

Pacific white shrimp (*Litopenaeus vannamei*) is an important aquacultured products favored worldwide, whose global production reached 5.8 million in 2020 [13,14]. After harvesting, chilling or freezing is often applied to retard the metabolic activities of microorganisms and endogenous protease hydrolysis. However, the hydrolysis and bacterial spoilage progress still move on even in chilling environment, leading to a limited shelf-life of several days [15]. However, besides the adverse effect of microorganisms and protein hydrolysis, melanosis is another essential problem to cause rejection from consumers. Melanosis occurs firstly when black spots appear on the cephalothorax of the shrimp, and finally develops thoroughly, mainly triggered by tyrosinase, which is also known as polyphenoloxidase (PPO), whose activity is related with catch/harvest season, gender, and size/age of specimens [16,17]. Although the precipitated melanin in shrimp is non-toxic, it can easily reduce the nutrient level, resulting in quality and economic loss [16]. Therefore, to extend the shelf life of shrimp, it is important to inhibit both the development of melanosis and the growth of bacteria. The inhibitory effect of some kinds of EOs on bacteria growth in seafood has been reported [7,9,10,18,19]. However, the studies about EOs on protein degradation and melanosis development of Pacific white shrimp still remains unclear.

In this study, the antibacterial and anti-melanosis activity of several essential oils (oregano essential oil (*Origanum vulgare*), wild orange essential oil (*Citrus sinensis*), tea tree essential oil (*Melaleuca Alternifolia*), and clove essential oil (*Eugenia caryophyllata*)) were evaluated both in vitro and *in situ*, so as to provide a whole view about the efficiency of the essential oils on the preservation of shrimp and propose a possible fresh-maintaining mechanism.

## 2. Materials and Methods

### 2.1. Chemicals

Oregano essential oil (OEO) and wild orange essential oil (WOEO) were obtained from döTERRA Shanghai Trading Co., Ltd. (Shanghai, China). Tea tree essential oil (TTEO) was obtained from Camenae Botanical Technology Co., Ltd. (Guangzhou, China). Clove leaf essential oil (CLEO) was brought from Ecoarts Enterprise Co., Ltd. (Shanghai, China). Trichloroacetic acid (TCA), sodium chloride, DPPH, and other chemicals were all obtained from Sinopharm chemical reagent company (Shanghai, China). All reagents were of the highest grade level available commercially. All microbial media were procured from Qingdao Hope Biol-Technology Co., Ltd. (Qingdao, China).

### 2.2. Bacterial Strains

*Staphylococcus aureus* (ATCC14222), *Escherichia coli* (ATCC25922), and *Bacillus cereus* (ATCC14579) were obtained from American Type Culture Collection (ATCC). *Shewanella putrefaciens* QY38 (NCBI accession no: KX692894) was isolated from spoiled Pacific white shrimps and stored in the laboratory previously.

### 2.3. In-Vitro Analysis of the Antioxidant and Antibacterial Ability

#### 2.3.1. Preparation of the Preservatives

The preservatives, including OEO, tea tree essential oil TTEO, wild orange essential oil WOEO, and clove leaf essential oil CLEO were diluted to 0.5%, 2%, and 10% in ethanol for antioxidant assays, respectively.

For the *in-vitro* antibacterial assay, the essential oils were diluted to 2.5%, 2%, 0.5%, 0.1%, 0.025%, and 0.005% (*v*/*v*), respectively, and sterilized by filtration through a 0.22 μm pore-size filter before use. The solvent ethanol was used as the control.

#### 2.3.2. Total Phenolic Content

Total phenolic content (TPC) of all the preservatives was determined as described by Olatunde, et al. [20] using gallic acid as standard.

#### 2.3.3. DPPH Scavenging Activity

DPPH scavenging activity of the preservatives was evaluated according to the method with some modifications [21]. The dilution of preservatives (25 μL) was mixed with 975 μL of DPPH ethanol solution (100 μmol/L), and then incubated at room temperature for 30 min in the dark. The absorbance of the solution was measured at 515 nm. The mixture of deionized water and DPPH ethanol solution was used as the blank, while the mixture of the preservatives and ethanol solvent was used as the control. The DPPH scavenging activity was calculated as follows:

(1) $$\mathrm{DPPH}\;\mathrm{scavenging}\;\mathrm{activity}\%=\left(1-\frac{A_{s}-A_{c}}{A_{b}}\right)\times 100\%$$

where *A_s_* represents the absorbance of the sample, *A_b_* represents the absorbance of blank, and *A_c_* represents the absorbance of the control.

#### 2.3.4. Oxford Cup Assay

These strains were activated by cultivation in Brain Heart Infusion (BHI) broth twice at 30 °C until the concentration reached approximately 10^8^ cells/mL. Then culture was diluted to about 10^6^ cells/mL by sterilized Luria-Bertani (LB) broth. Then the Oxford cup assay was conducted according to Qian, Cheng, Ye, Zhao, Xie and Yang [18]. Finally, the diameter of the inhibition zone was measured after incubation at 30 °C for 24 h.

### 2.4. In-Situ Study on the Effect of the Selected Preservatives on the Qualities of Pacific White Shrimp

#### 2.4.1. Sample Preparation

Pacific white shrimp samples (16–20 g for each) were purchased from local market near Luchaogang Port (Shanghai, China) alive in water (14–18 °C) within 30 min. Upon arrival at the laboratory, the shrimps were immersed into the ice-slurry and washed. Then the shrimp was drained and separated into 4 groups randomly.

#### 2.4.2. Preparation and Application of Preservatives

Oregano essential oil (OEO) and clove leaf essential oil (CLEO) were used to preserve Pacific white shrimp during cold storage. According to the *in-vitro* antioxidant and anti-bacterial experiment, the two essential oils were diluted to 2% (*v*/*v*) in water. A compound preserving solution was prepared with 2% OEO and 2% CLEO at a ratio of 1:1. Then the shrimp were immersed into the essential oils for 5 min before draining. The four treatments are listed as following: (1) Con: treated by sterilized water; (2) OEO: treated by 2% OEO; (3) CLEO: treated by 2% CLEO; (4) OEO + CLEO: treated by 1% OEO + 1% CLEO.

#### 2.4.3. Microbiological Analysis

The total viable counts, psychrotrophic bacteria, and H_2_S-producing bacteria growth were measured according to Yu, et al. [22]. In brief, 25 g of shrimp was homogenized with 225 mL sterilized saline water (0.85%, *w*/*v*), and diluted in serial 10-fold solutions. One milliliter of the dilutions was mixed with Plate count agar (PCA, No.HB0101, Qingdao Hope Biol-Technology Co., Ltd., Qingdao, China) or Iron agar (IA, NO. HB8735, Qingdao Hope Biol-Technology Co., Ltd., Qingdao, China). For total viable counts and psychrotrophic bacteria counts, the plates with PCA were incubated at 30 °C for 48 h and 4 °C for 10 days, respectively. For H_2_S-producting bacteria counts, the plates with IA were incubated at 25 °C for 72 h, and the black colonies were counted for calculation.

#### 2.4.4. Total Volatile Basic Nitrogen (TVB-N)

The TVB-N content of shrimp was detected by using the steam-distillation procedure on an Automatic Kjeldahl Apparatus (Kjeltec Analyzer Unit, Foss Tecator AB; Hoganas, Sweden) [22]. The results were recorded as mg N/100 g. The procedure was repeated in triplicate.

#### 2.4.5. Weight LOSS

Weight loss (%) was evaluated by weighing shrimp during storage and evaluated by the following equation according to the reference [23].

(2) $$\mathrm{Weight}\;\mathrm{loss}\;\left(\%\right)=\frac{m_{0}-m_{n}}{m_{0}}\times 100$$

where *m*_0_ was the weight of shrimp at the beginning of storage, and *m_n_* was the weight of shrimp after *n* days of storage.

#### 2.4.6. Determination of TCA-Soluble Peptides Content

Trichloroacetic acid (TCA)-soluble peptides contents were determined according to the method of Chen, et al. [24].

#### 2.4.7. Determination of Sarcoplasmic and Myofibrillar Protein Contents

The protein was extracted according to the method of Lv, et al. [25]. About 2 g minced fish sample was homogenized with 20 mL Tris-maleate buffer A (20 mmol/L pH 7.0, 0.05 mol/L KCl) at 4 °C. Then the solution was centrifuged for 15 min at the speed of 10,000× *g.* The supernatant containing sarcoplasmic protein was collected. The precipitate was homogenized with 20 mL Tris-maleate buffer B (20 mmol/L, pH 7.0, containing 0.6 mol/L KCl). The solution was incubated for 1 h at 4 °C. After centrifugation at 10,000× *g* for 15 min, the supernatant containing myofibrillar protein was obtained. The protein content of the extracted solution was determined by the BCA Protein Assay Kit (Tiangen Biotech (Beijing) Co., Ltd., Beijing, China) and the results were expressed as mg/mL of the extraction.

#### 2.4.8. Colorimetric Measurement

The surface color including L* (lightness), a* (redness and greenness), b* (yellowness and blueness) of shrimp cephalothoraxes were measured via YS6060 Benchtop Grating Spectrophotometer (Shenzhen Threenh Technology Co., Ltd., Shenzhen, China) in accordance with the method described by Qian, et al. [26]. The whiteness value was calculated according to Equation (3), and then the mean values of samples per group were calculated.

(3) $$\mathrm{Whiteness}=100-\sqrt{{\left(100-L^{*}\right)}^{2}+{\left(a^{*}\right)}^{2}+{\left(b^{*}\right)}^{2}}$$

#### 2.4.9. Melanosis Ratio

The photos of the shrimp samples were loaded to Adobe Photoshop 2020 (Adobe Systems Incorporated, San Jose, CA, USA). After eliminating the background, the black area was identified using the Magic Wand Tool with a tolerance of 4%, and the black pixels and the pixels of the whole shrimp were calculated. The melanosis ratio of the black pixels to the total pixels of the whole shrimp was used to evaluate the development of melanosis (Equation (4)).

(4) $$\mathrm{Melanosis}\;\mathrm{ratio}\%=\frac{\mathrm{Pixels}\;\mathrm{of}\;\mathrm{black}\;\mathrm{area}}{\mathrm{Total}\;\mathrm{pixels}\;\mathrm{of}\;\mathrm{the}\;\mathrm{whole}\;\mathrm{shrimp}\;\mathrm{area}}\times 100\%$$

#### 2.4.10. Polyphenol Oxidase Activity

The polyphenol oxidase activity was determined using the polyphenol oxidase kit (Nanjing Jiancheng Bioengineering Institute, Nanjing, China). The shrimp (0.1 g) was homogenized with 1 mL extraction buffer. Then the extraction solution was mixed with the substrate solution, and the absorbance was measured via a spectrophotometer (752 N, Shanghai INESA Scientific Instrument Co., Ltd., Shanghai, China) at 420 nm. One unit (1 U) of PPO activity was defined as an increment of 0.01 absorbance/min. The PPO activity of the sample was expressed as specific activity in units of enzyme activity per mg protein (U/mg protein).

### 2.5. Statistical Analysis

All experiments were conducted at least in triplicate. The one-way ANOVA procedure followed by Duncan’s test was used by the SPSS software (*p* < 0.05, SPSS Version 20.0, Inc., Chicago, IL, USA), and the results were expressed as means ± SD. The diagrams were designed by the Origin2018 software (OriginLab, Northampton, MA, USA).

## 3. Results and Discussion

### 3.1. In-Vitro Analysis of the Antioxidant and Antibacterial Ability

#### 3.1.1. Total Phenolic Content

The total phenolic contents of the preservatives are shown in Table 1. It is known that polyphenols are a kind of bioactive compounds, which are considered to be responsible for the antibacterial and antioxidant activity [27]. The results showed that the TPC of OEO, TTEO, and CLEO were close to each other, while the TPC of WOEO was much lower. This phenomenon should be related to the different predominant compounds in these essential oils. The predominant component in WOEO was limonene, belonging to terpene groups, and other essential oils had higher contents of phenol compounds.

#### 3.1.2. DPPH Scavenging Activity

Melanosis begins from the oxidation of tyrosine triggered by tyrosinase [16], therefore an antioxidant is considered to have a potential ability to prevent melanosis. The antioxidative capacities of the preservatives were determined by the DPPH free radical scavenging assay, and the results are also displayed in Table 1. Generally, the DPPH scavenging activity of the essential oils exhibited a dose-dependent relationship at different extents. The DPPH scavenging activity of CLEO was significantly higher than other essential oils. The results of OEO were close to the study by Kosakowska et al. [28], which contributed the DPPH scavenging activity of OEO to carvacrol and/or thymol. It is reported that the free radical scavenging activity of CLEO is attributed to the phenylpropanoids such as eugenol and its derivatives [29]. The results confirmed that having the same level of phenolic content does not necessarily mean the same antioxidant response [30]. It is reported that the antioxidant effect relates with the hydrophilic-lipophilic balance of the extract and the chemistry of the surrounding environment [31]. Therefore, the antioxidant activity of EOs is also related to the solvent polarity [28]. To scavenge 50% DPPH, the concentrations of OEO and CLEO should not be less than 2% and 0.5%, respectively.

#### 3.1.3. Oxford Cup Assay

The sensitivity of the four bacterial species to the four essential oils is shown in Table 2. In general, all preservatives showed promising antibacterial properties against the selected bacteria. The dimensions of the inhibition halos increased with the increasing concentrations of essential oils, but the sensitiveness differed from the bacterial species and the oils used. The minimum inhibitory concentration (MIC) is defined when the diameters of inhibition halos were higher than 10 mm. According to the results, the minimum inhibitory concentrations (MICs) of oregano essential oil against *S. aureus*, *B. subtilis*, *S. putrefaciens*, and *E. coli* were 0.1%, and the MIC of TTEO was 10%, and that of CLEO was about 0.5%. The MIC of OEO was close to the previous study [28]. WOEO had the highest MICs than other oils, indicating the lowest antibacterial activity. The antibacterial activity of these essential oils could be contributed to the polyphenolic compounds, which can interact with bacterial cell membrane due to the hydrophobic/lipophilic property, consequently leading to the destruction of membrane and leakage of cell components [32,33].

Among these selected plant essential oils, OEO and CLEO displayed a better antioxidant and antibacterial effect. Considering the low adhesion rate into shrimp, the 4MICs should be applied for further *in-situ* study. Therefore, the concentrations of OEO and CLEO applied in Pacific white shrimp for further study were 2%.

### 3.2. In-Situ Study on the Effect of the Selected Essential Oils on the Quality of Pacific White Shrimp

#### 3.2.1. Microbiological Growth

**Figure 1:**
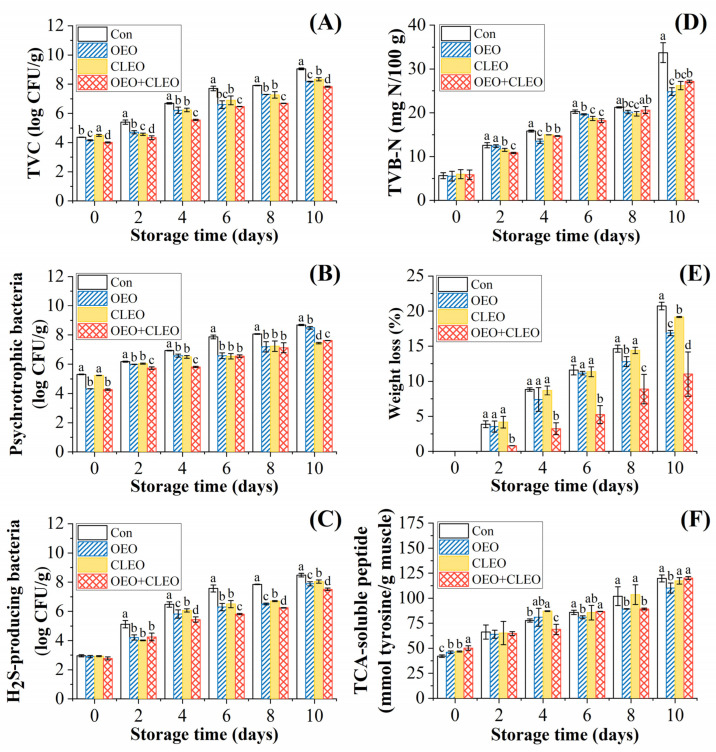
Changes of total viable counts (**A**), psychrotrophic bacterial counts (**B**), H_2_S-producing bacterial counts (**C**), TVB-N (**D**), weight loss (**E**) and TCA-soluble peptide (**F**) of refrigerated Pacific white shrimp treated with OEO and CLEO at 4 °C. Data are shown as mean ± SD (n = 3). Note: Different lowercase letters indicate significant differences (*p* < 0.05) among different treatments within the same storage time.

The changes of total viable counts, psychrotrophic bacterial counts, and H_2_S-producing bacterial counts of shrimp treated with essential oils are displayed in Figure 1A–C. The initial total viable counts (TVCs) of the four groups were close to each other, i.e., 4.37 log CFU/g, 4.16 log CFU/g, 4.50 log CFU/g, and 4.01 log CFU/g, respectively (Figure 1A). The total viable counts of all groups were all increased during storage. The essential oil treatment group was significantly lower than the control group (*p* < 0.05). It is illustrated that the essential oils had an obvious effect of bacteriostasis, which was consistent with the results of the *in-vitro* experiments mentioned above. The phenolic compounds such as carvacrol in OEO and eugenol in CLEO should be attributed to the antibacterial effect [34]. The TVC of samples treated with OEO or CLEO only was close to each other, indicating that they had a similar antibacterial effect, but was slightly higher than the combined treatment. The control exceeded the threshold of 7 log CFU/g [35] on day 6 (7.71 log CFU/g), while the sample treated by OEO + CLEO reached the limitation on day 10 (7.83 log CFU/g). Therefore, the shelf life of shrimp was prolonged by about 4 days by OEO + CLEO. It was demonstrated the combination of the two essential oils could enhance the effect of bacteriostasis.

The initial counts of psychrotrophic bacteria of shrimp were higher than TVC, and also increased during the whole storage period (Figure 1B). Therefore, the spoilage of shrimp during refrigerated storage was mainly attributed to the proliferation of psychrotrophic bacteria. During the storage period, the samples treated with essential oils were always lower than the control group, indicating its inhibitory effect on the growth of psychrotrophic bacteria.

The changes of H_2_S-producing bacterial counts of Pacific white shrimp during storage are shown in Figure 1C. *Shewanella* species, including *S. putrefaciens* and *S. baltica* are reported to be the predominant H_2_S-producing bacteria in shrimp, which has the capacity to produce trimethylamine, H_2_S, putrescine, and other off-odor compounds [36]. The initial counts of H_2_S-producing bacteria in the four groups were close to each other, but the increase in samples treated with essential oils was retarded. At the end of storage, the counts of the four groups increased to 8.48 log CFU/g, 7.89 log CFU/g, 8.04 log CFU/g, and 7.51 log CFU/g, respectively. CLEO displayed a slightly lower effect of bacteriostasis than OEO, in accordance with the *in-vitro* measurement. However, the combination of the two essential oils showed the highest antibacterial effect, indicating that there was a synergistic effect.

#### 3.2.2. Total Volatile Basic Nitrogen (TVB-N)

The TVB-N value reflects the amount of trimethylamine, dimethylamine, ammonia and other amines produced by the decomposition of protein in aquatic products under the combined action of microorganisms and enzymes [37]. The changes of TVB-N value of Pacific white shrimp treated with essential oils during storage are shown in Figure 1D. The initial TVB-N value of shrimp was about 5.52~5.85 mg N/100 g, but increased to 33.73 mg N/100 g, 24.83 mg N/100 g, 26.21 mg N/100 g, and 27.15 mg N/100 g at the end of storage, respectively. The contents of TVB-N of the control during storage were close to the study previously reported [18]. The TVB-N values of shrimp treated with essential oils were significantly lower than the control after 10 days of storage.

#### 3.2.3. Weight Loss

Figure 1E showed the weight loss of Pacific white shrimp treated with essential oils during storage. The weight loss is mainly related with the drip loss of muscle, due to the decomposition, denaturation, or oxidation of proteins [38,39]. The changes of weight loss were indeed positively correlated with changes of bacterial growth, TCA-soluble peptide, and negatively correlated with protein contents displayed as following in this study. Therefore, the capacity to prevent weight loss of products is an important indicator to evaluate the efficiency of preservatives. The results showed that all samples treated with essential oils had lower weight loss than the control. Among the three treated samples, the samples treated with OEO + CLEO had the lowest weight loss, indicating that OEO + CLEO could inhibit the changes of protein effectively.

#### 3.2.4. Changes of TCA-Soluble Peptide Content

The content of TCA-soluble peptide in shrimp increased during the storage period (Figure 1F), which was negatively correlated with the changes of sarcoplasmic and myofibrillar proteins. The increase of TCA-soluble peptide should be attributed to the degradation of proteins triggered by endogenous enzymes and contaminated bacteria [40]. Therefore, the higher the amount of TCA-soluble peptide means the greater the degradation of the protein. Compared with the four groups, it was found that OEO and OEO + CLEO displayed a better effect on inhibiting the increase of TCA-soluble peptide.

#### 3.2.5. Changes of Sarcoplasmic and Myofibrillar Contents

**Figure 2:**
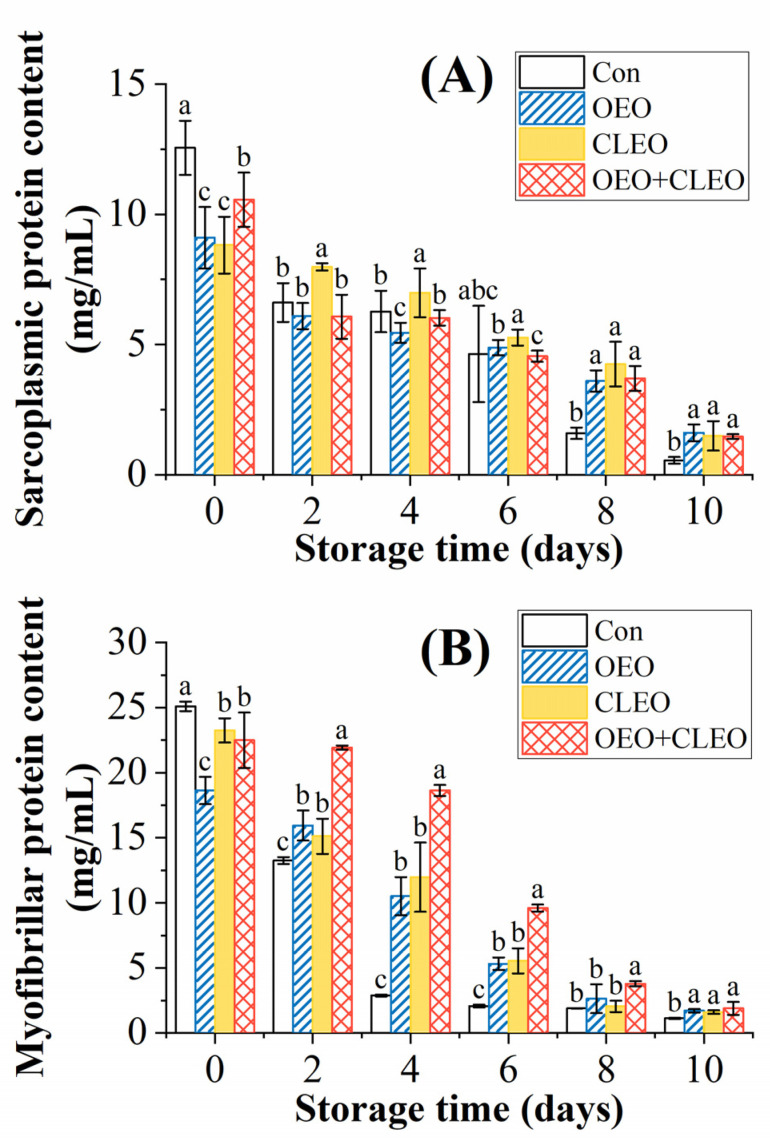
Changes of the content of sarcoplasmic protein (**A**) and myofibrillar protein (**B**) of refrigerated Pacific white shrimp treated with OEO and CLEO at 4 °C. Data are shown as mean ± SD (n = 3). Note: Different lowercase letters indicate significant differences (*p* < 0.05) among different treatments within the same storage time.

The muscle protein of shrimp mainly consists of the sarcoplasmic and myofibrillar proteins, which are water-soluble and salt-soluble protein, respectively [41]. As shown in Figure 2A,B, the contents of sarcoplasmic and myofibrillar protein of shrimp decreased during storage, which were negatively correlated with the changes of bacterial gowth, TCA-soluble peptide, and TVB-N. The initial contents of sarcoplasmic protein of shrimp were 12.56 mg/mL, 9.10 mg/mL, 8.82 mg/mL, and 10.56 mg/mL on 0 d, respectively. It is reported that the sarcoplasmic protein is mainly composed by proteases and other low-molecular proteins [42]. The control group was generally lower than the treatment group, especially after 8 days of storage (*p* < 0.05). It is illustrated that the treatment with essential oils could significantly delay the protein degradation of Pacific white shrimp.

The myofibrillar protein is regarded as the main muscle protein in shrimp, which is composed of salt soluble proteins such as myosin, actin, and troponins [41]. The initial contents of myofibrillar protein in shrimp treated with essential oils were 18.63 mg/mL, 23.25 mg/mL, and 22.50 mg/mL, respectively, which were slightly lower than the control (25.10 mg/mL). The content of myofibrillar protein of the control decreased significantly in the first 4 days and then maintained at a low level till the end of storage. It was found that the essential oils could retard the decreasing rates of the myofibrillar protein, which should be attributed to their antibacterial activity. The samples with the combined treatment had the highest content of myofibrillar protein, indicating the efficiency to preventing protein degradation.

#### 3.2.6. Colorimetric Measurement

**Figure 3:**
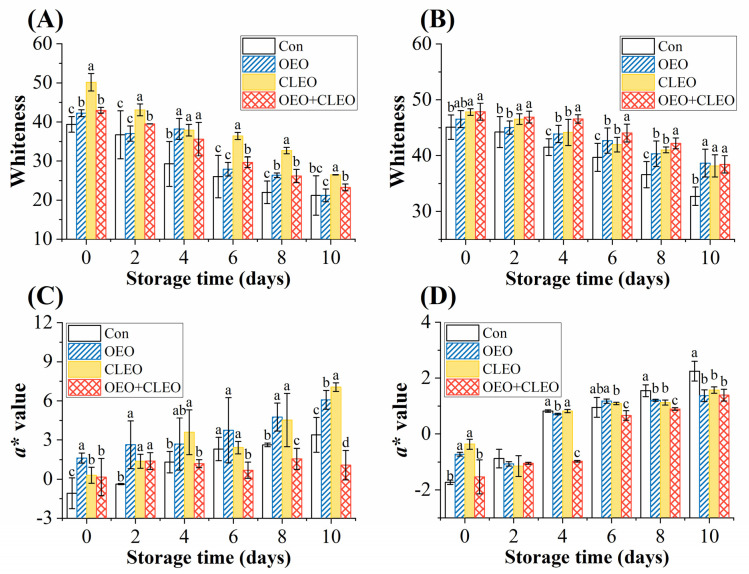
Changes of whiteness and a* of cephalothorax (**A**,**C**) and abdomen (**B**,**D**) of refrigerated Pacific white shrimp treated with OEO and CLEO at 4 °C. Data are shown as mean ± SD (n = 3). Note: Different lowercase letters indicate significant differences (*p* < 0.05) among different treatments within the same storage time.

The whiteness of cephalothorax and abdomen of Pacific white shrimp are observed in Figure 3A,B. The whiteness value of all groups showed a decreasing tendency during storage. The shrimp treated with essential oils had the higher whiteness values than the control, especially the samples treated with CLEO and OEO + CLEO. At the end of storage, the whiteness values of shrimp cephalothorax of the four groups were 21.19, 21.20, 26.48, and 23.25, respectively, and the whiteness values of the abdomen were 32.71, 38.67, 38.16, and 38.44. The anti-melanosis effect of essential oils was also confirmed by the results. The CLEO was more helpful to maintain the whiteness value of shrimp cephalothorax, due to its higher antioxidant activity.

The redness (a*) value of shrimp cephalothorax and abdomen both increased during the storage (Figure 3C,D). The increase of a* value of shrimp cephalothorax was much faster than that of the shrimp abdominal section. The samples treated with OEO + CLEO were lower than other groups. The increase of redness value could be related with the decomposition of astaxanthin-protein complexes and the release of astaxanthin monomers [43].

#### 3.2.7. Melanosis Ratio

**Figure 4:**
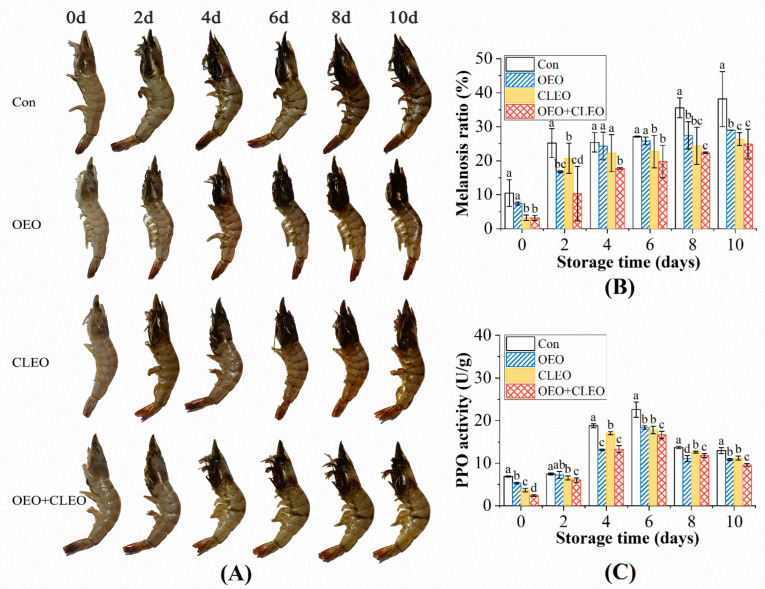
Photograph (**A**) and changes of melanosis ratio (**B**) and PPO activity (**C**) of refrigerated Pacific white shrimp treated by OEO and CLEO at 4 °C. Data are shown as mean ± SD (n = 3). Note: Different lowercase letters indicate significant differences (*p* < 0.05) among different treatments within the same storage time.

The overall photographs of Pacific white shrimp during storage are shown in Figure 4A. The melanosis area ratio was calculated accordingly to evaluate the development of melanosis all over the shrimp sample, and the results are shown in Figure 4B. The melanosis usually starts from the cephalothorax and then spreads to the abdomen and the tail [44]. The melanosis ratio of the control was significantly higher than the treated samples throughout the storage period, indicating more black spots occurred on the whole shrimp. The melanosis ratio was negatively correlated with the changes of whiteness values. CLEO seemed to have a stronger inhibitory effect on melanosis than OEO, as the shrimp treated with OEO had a higher melanosis ratio. This might be contributed to the higher antioxidant activity of CLEO as demonstrated in the DPPH scavenging activity. The combined treatment of OEO and CLEO displayed the lowest melanosis ratio, indicating that the two essential oils had a synergetic effect on inhibiting the spread of black spots.

#### 3.2.8. PPO Activity

PPO is an important enzyme involved in a variety of physiological activities in crustaceans, which catalyzes the oxidation of tyrosine and undergoes several reactions to form dark pigments [44]. The changes in the PPO activities of shrimp during storage are shown in Figure 4C. The initial PPO activity value of the samples treated with essential oils were 5.30 U/g, 3.69 U/g, and 2.40 U/g, which were lower than the control group (6.87 U/g). The result indicated that essential oils could inhibit the PPO activity immediately after treatment. The PPO activity of all groups went up to the peak on 6 d, and then decreased to some extent. The decreasing tendency of PPO activity should be contributed to proteolytic degradation by endogenous and bacteria protease. Similar phenomenon was also illustrated in other literatures [45]. The PPO activities of treated samples were 18.37 U/g, 17.79 U/g, and 16.64 U/g on day 6, respectively, while the control was about 22.57 U/g. The CLEO displayed a higher inhibitory effect on PPO activity than OEO, which was in accordance with their DPPH scavenging activity. It is hypothesized that phenolic compounds in essential oils inhibit the activity of PPO by competing with the substrates [46]. Moreover, the combination of the two essential oils had the highest efficiency. The increasing tendency of PPO activity in the first period was corresponded with the changes of the melanosis ratio and whiteness value.

### 3.3. Schematic Illustration

**Figure 5:**
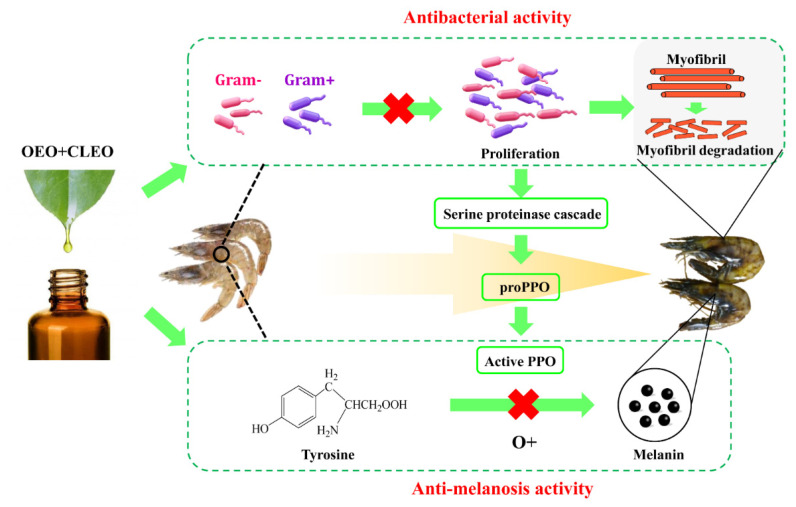
Schematic diagram of the antibacterial and anti-melanosis activity of OEO + CLEO on Pacific white shrimp during cold storage.

A schematic illustration of the effect of essential oils on melanosis and quality changes of Pacific white shrimp was proposed (Figure 5). The effect of OEO + CLEO to maintain the quality of Pacific white shrimp should basically contributed to their antibacterial and antioxidant activity as illustrated in the *in-vitro* study, and consequently inhibiting the quality deterioration related with bacterial growth and oxidation reactions. During cold storage, the bacteria proliferated and induced the degradation of muscle protein, leading to the decrease of protein content and increase of TCA-soluble peptides and TVB-N. In this study, the effect of OEO + CLEO on retarding the deterioration process due to antibacterial process was proved. Regarding to the melanosis development, it is known that the melanin is produced from tyrosine triggered by PPO in the presence of oxygen [47]. Therefore, inhibitors which can inhibit the activity of PPO or scavenge free oxygen can be used to as anti-melanosis agents [48]. OEO and CLEO deprived from plants, are known to be rich in phenolic compounds, and they could probably inhibit melanosis by inhibit PPO activity and scavenging free oxygen. On the other hand, PPO is known to exist in crustaceans as zymogens and plays a key role in the primary immune response system, and it can be activated because of the serine proteinase cascade triggered by microbial compounds (carbohydrates and lipopolysaccharides) and a series of other proteins [45,47,49]. Therefore, there could be another possible way for the essential oils to inhibit melanosis by retarding the proliferation of bacteria. A relationship between bacteria and melanosis was also displayed in the diagram.

## 4. Conclusions

This work describes the ability of essential oil treatment to extend the shelf life of Pacific white shrimp. The antioxidant and antibacterial efficiency of the four essential oils were screening via *in-vitro* experiment. It was found that CLEO and OEO had higher antioxidant and antibacterial effect than WOEO and TTEO. Therefore, CLEO and OEO were applied to preserve shrimp for further *in-situ* study. The results indicated that both CLEO and OEO could inhibit the quality deterioration of shrimp effectively. The CLEO displayed higher antioxidant activity, as well as a higher inhibitory effect on PPO activity and melanosis, while OEO had higher effect on preventing the increase of bacterial growth, TVB-N value, weight loss, and TCA-soluble peptides. This study highlighted that the combination of CLEO and OEO enhanced their antioxidant and antibacterial effect, and could maintain the quality of shrimp most effectively, consequently prolonging the microbiological shelf-life by about 4 days. Therefore, this study expanded the usage of EOs in Pacific white shrimp, and the convenience of this method made it to be a potential use for industrial application.

## Figures and Tables

**Table 1:** Total phenolic content (TPC) and DPPH scavenging activity (%) of different preservatives.

	Concentration (*v*/*v*)	OEO	TTEO	WOEO	CLEO
TPC (mg GAE/100 g)	10%	20.97 ± 0.22	20.29 ± 0.35	13.34 ± 0.51	21.12 ± 0.01
DPPH scavenging activity (%)	0.5%	42.28 ± 1.15	22.76 ± 1.15	27.64 ± 1.15	79.67 ± 1.15
	2%	73.11 ± 0.54	28.22 ± 0.27	54.55 ± 0.54	84.85 ± 0.54
	10%	84.55 ± 3.45	61.79 ± 2.30	65.85 ± 1.15	87.80 ± 2.30

Note: oregano essential oil (OEO), tea tree essential oil (TTEO), wild orange essential oil (WOEO), and clove leaf essential oil (CLEO).

**Table 2:** The sensitivity of the four bacterial species to the four essential oils.

Bacterium	Presser-Vatives	Concentrations (%)						
		10	2.5	0.5	0.1	0.025	0.005	Negative Control
*S. aureus* (+)	OEO	++++	++++	++++	++	+	−	−
	TTEO	++	+	+	−	−	−	−
	WOEO	++	+	+	−	−	−	−
	CLEO	++++	+++	++	+	+	−	−
*B. subtilis* (+)	OEO	++++	++++	++++	++	+	−	−
	TTEO	++	++	+	−	−	−	−
	WOEO	+	+	−	−	−	−	−
	CLEO	++++	+++	++	+	+	−	−
*S. putrefaciens* (−)	OEO	++++	++++	++++	++	+	+	−
	TTEO	++	++	+	−	−	−	−
	WOEO	+++	++	+	−	−	−	−
	CLEO	++++	++++	++	+	+	+	−
*E. coli* (−)	OEO	++++	++++	++++	++	−	−	−
	TTEO	++	+	−	−	−	−	−
	WOEO	+	+	−	−	−	−	−
	CLEO	++++	+++	++	+	+	+	−

Note: “++++” means extremely sensitive (bacteriostatic diameter ≥ 20 mm); “+++” means quite sensitive (15 mm ≤ bacteriostatic diameter < 20 mm); “++” means sensitive (10 mm ≤ bacteriostatic diameter < 15 mm); “+” means slightly sensitive (8 mm < bacteriostatic diameter < 10 mm); “−” means not sensitive (bacteriostatic diameter ≤ 8 mm).

## Data Availability

The datasets for this study can be found in https://figshare.com/s/4a4358daa3054f71b4ed (accessed on 1 February 2022).
